# Therapeutic potential of tumor-associated neutrophils: dual role and phenotypic plasticity

**DOI:** 10.1038/s41392-025-02242-7

**Published:** 2025-06-04

**Authors:** Yanting Zhou, Guobo Shen, Xikun Zhou, Jing Li

**Affiliations:** 1 State Key Laboratory of Oral Diseases, National Clinical Research Center for Oral Diseases, Chinese Academy of Medical Sciences Research Unit of Oral Carcinogenesis and Management, West China Hospital of Stomatology, Sichuan University, Chengdu, Sichuan China; 2 Department of Biotherapy, Cancer Center and State Key Laboratory of Biotherapy, West China Hospital, Sichuan University, Chengdu, China; 3 Laboratory of Pathogen Research, West China Hospital, Sichuan University, Chengdu, China

**Keywords:** Cancer microenvironment, Tumour immunology, Cancer microenvironment

## Abstract

Neutrophils are the first line of defense in nonspecific immunity (innate immunity) and interact with other immune cells to participate in specific defense mechanisms (adaptive immunity). Studies have shown that the tumor microenvironment (TME) mediates tumor development and recruits neutrophils into tumors to become tumor-associated neutrophils (TANs), an important part of TME, and achieve extended lifespan. TANs can be differentiated into the antitumor or protumor phenotype, and play an important role in tumor occurrence, proliferation and recurrence, invasion and metastasis, angiogenesis, cell necrosis, and so on. Here, we summarize the TAN origin and subtypes found through Single-cell RNA sequencing analysis in different types of tumors in recent literature, and the molecular mechanisms underlying their antitumor and protumor effects on tumors. We focus on the interaction between TANs and immunosuppressive or immunostimulatory TME, as well as signal pathways such as transforming growth factor β (TGF-β) associated with TAN phenotype transition. Based on the summarized mechanisms, we focus on the potential application and latest strategies of TAN-based immunotherapy, chemotherapy, and combination therapy in the preclinical study and clinical trials of tumors. The discussion on promising therapy encompasses five key areas: inhibition of the tumor-promoting effect of TANs, enhancement of the antitumor effect of TANs, targeting the interaction between TANs and the TME, reprogramming of TANs, and drug delivery carriers. Finally, we discuss the potential of TANs and their related markers as emerging biomarkers for predicting the prognosis of cancer patients.

## Introduction

**Fig. 1:**
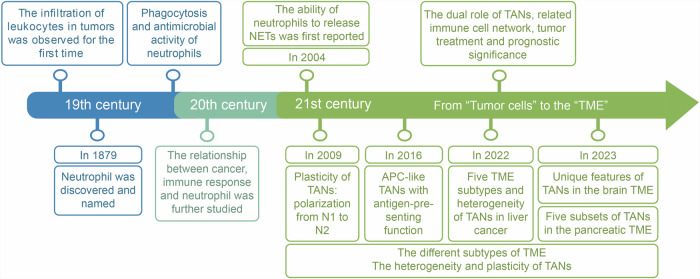
History of tumor-associated neutrophils. With the development of technology, research on TANs has gradually improved. In the 19th century, the pathologist Rudolf Virchow first observed leukocyte infiltration in tumors.^12^ Subsequently, Paul Ehrlich discovered and officially named “neutrophils” in 1879.^10^ Elie Metchnikoff also demonstrated the phagocytosis and antibacterial activity of neutrophils.^11^ Research on the relationship between neutrophils and the tumor immune response has gradually increased.^13,14^ In 2004, the phenomenon of neutrophil NETosis was discovered.^25^ In 2009, the theory that TANs are polarized into N1 and N2 phenotypes was proposed, accompanied by the discovery of other phenotypes of TANs.^2^ For example, antigen-presenting cell (APC)-like TANs were proposed in 2016.^45^ At the same time, scientists have focused on the important connection between TANs and the TME. Since 2022, scientists have studied many different TMEs and unique types of TANs.^34,38,42^ However, the dual role and clinical applications of TANs still require further research. Figures were created with Adobe Illustrator

The tumor microenvironment (TME), which comprises tumor cells, immune cells, stromal cells, and extracellular matrix, is reshaped by tumor-associated neutrophils (TANs) and induces a distinctive phenotype in TANs in turn. The specificity of intratumoral localization, the diversity of cytokines, and other local conditions (such as hypoxia, inflammation, and high levels of lactic acid^1^) within the matrix of the TME is highly informative. Since the antitumor and protumor phenotypes of TANs were first named with N1 TANs and N2 TANs^2^ (Fig. 1), respectively, the N1-N2 concept has been widely adopted due to its concise description of functional dichotomy, though primarily in murine models. It is generally believed that TANs exhibit an antitumor N1 phenotype at the edge of the tumor during the early stages, whereas they are gradually recruited to the tumor core during later stages and subsequently reprogrammed to an immunosuppressive N2 phenotype.^3,4^ Recent studies have demonstrated that N1 TANs with elevated levels of intercellular adhesion molecule 1 and CD95 (Fas/Apo1) expression infiltrate the invasive margin of early-stage human colorectal cancer (CRC).^5^ In mice, N1 TANs exhibit hypersegmented nuclei, while N2 TANs possess circular nuclei and can be distinguished morphologically.^2,6^ Interferon (IFN) and other factors drive the polarization of TANs toward the N1 type, featuring high expression of major histocompatibility complex class II and costimulatory molecules.^7^ Conversely, factors like transforming growth factor β (TGF-β) stimulate the formation of N2 TANs, which highly express arginase 1 (ARG1) and some chemokine (C-C motif or C-X-C motif) ligands, including CCL2, IL-8/CXCL8.^2^ Although in many tumors (such as CRC and lung adenocarcinoma), the number of TANs is low in the leukocyte subsets,^8^ research on TANs has increased significantly due to their therapeutic potential, which has been increasingly recognized, and their close relationship with the prognosis of cancer patients in recent years. TANs have both antitumor and protumor effects in cancer and display phenotypic and functional diversity and plasticity by interacting with the matrix and cells (such as tumor-associated macrophages (TAMs), T cells, and natural killer (NK) cells) in the TME.^9^

Here, we review the specific mechanisms by which TANs with different phenotypes promote and inhibit tumors. We explored their interactions with the TME, the reprogramming of TANs, and their value in predicting tumor prognosis. We summarize the potential applications of TANs in tumor therapy and currently emerging tumor-targeted treatment strategies.

## History and progression of tumor-associated neutrophils

**Fig. 2:**
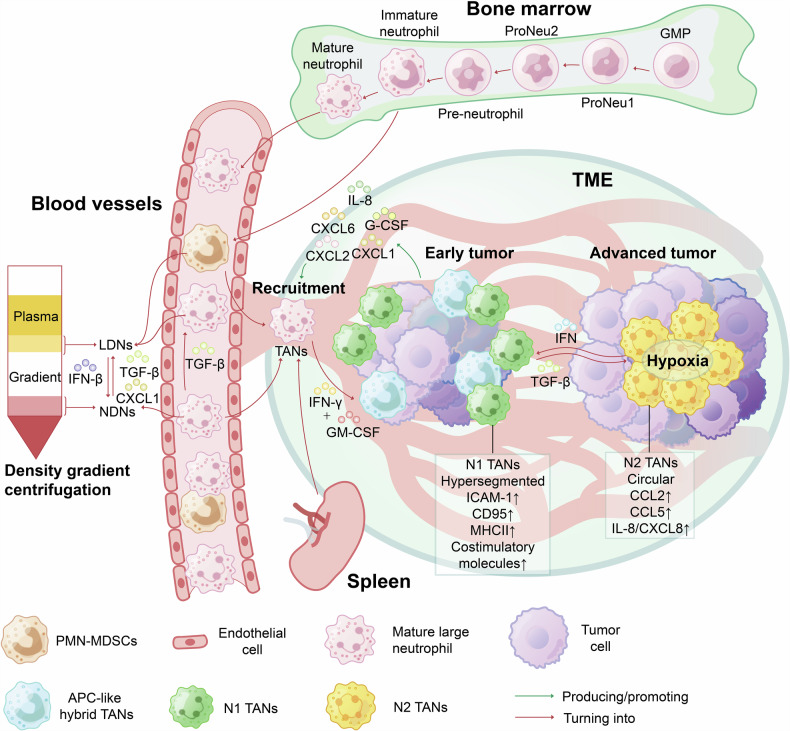
Two main phenotypes of tumor-associated neutrophils within the tumor microenvironment. Neutrophils undergo a series of developmental stages in the bone marrow, including granulocyte–monocyte progenitors (GMPs), proneutrophil stage 1 (proneu1), proneutrophil stage 2 (proneu2), and immature neutrophils, and ultimately culminate in mature neutrophils. Following this maturation process, both mature neutrophils and a small number of immature neutrophils enter the circulation, subsequently developing into PMN-MDSCs, large neutrophils, and small neutrophils. The former two can be categorized as LDNs based on density gradients, whereas the latter may be classified as NDNs. In response to a variety of tumor cell-derived chemokines, such as CXCL1, CXCL2, CXCL6, IL-8, and G-CSF, circulating neutrophils can be recruited to the TME and become TANs, where they can differentiate into various phenotypes. The reduction in the number of protumor TANs recruited can pave the way for improved prognostics in cancer therapy. Typically, TANs at the edge of the tumor during the early stages exhibit an antitumor N1 phenotype, while those gradually mobilized to the tumor core in the later stages display an immunosuppressive N2 phenotype. N1 TANs express relatively high levels of ICAM-1 and CD95, exhibit hypersegmented nuclei, and are associated with high MHCII and costimulatory molecule expression. In contrast, N2 TANs possessed round nuclei with high expression of CCL2, CCL5, IL-8, and ARG1. Nevertheless, other TAN phenotypes, such as APC-like TANs, which are closely associated with IFN-γ and GM-CSF levels and hypoxia conditions in the TME, have also been identified (ICAM-1 intercellular adhesion molecule 1, MHCII major histocompatibility complex class II.)

In 1879, Paul Ehrlich discovered and named “neutrophils”,^10^ which Elie Metchnikoff proved to exhibit phagocytosis behavior and antibacterial effects^11–14^ (Fig. 1). The neutrophil developmental process involves common myeloid progenitors, granulocyte–monocyte progenitors, promyelocytes, human myeloid cells, band neutrophils, and segmental neutrophils, eventually differentiating into mature neutrophils^15–17^ (Fig. 2). Mature neutrophils are generally reported to remain in the bone marrow (90%) for 4–7 days and can exist in the blood (1–5%) with a relatively short half-life.^18^ In mice and humans, neutrophil circulation numbers and tissue infiltration exhibit circadian rhythms corresponding to the respective species’ activity cycles.^19^

Neutrophils follow pathogen-associated molecular patterns (PAMPs) and damage-associated molecular patterns (DAMPs) to complete the recruitment cascade to the infection or injury site through a series of steps, including rolling, adhesion, intravascular crawling, and migration into tissues.^20^ Neutrophils mediate nonspecific defense (innate immunity) by phagocytosing pathogens,^21^ degranulation (the release of particles containing cytotoxic factors, such as antibacterial compounds, serine endopeptidase, lysozyme, etc.),^22^ the expulsion of neutrophil extracellular traps (NETs), the release of reactive oxygen species (ROS) and reactive nitrogen species (RNS), and the regulation of immune signaling pathways.^23^ NETs are extracellular web-like structures composed of DNA, histones, and cytotoxic granule proteins such as myeloperoxidase (MPO), neutrophil elastase (NE), and cathepsin G (CG), released by neutrophils.^24^ First described in 2004, NETs have been shown to capture and kill microorganisms^25^ (Fig. 1).

Neutrophils with high levels of chemokine receptors are induced to migrate by chemokine ligands (such as interleukin (IL)-8^26^ and granulocyte colony-stimulating factor (G-CSF)^27^) that are secreted by tumor cells,^28^ stromal cells, or other immune cells to form circulating neutrophils outside the bone marrow.^29^ They are further recruited to the TME by a “chemokine storm” to form TANs, known as chemotaxis^30^ (Fig. 2). Neutrophils are typically the first cells to reach early-stage neoplastic sites.^31,32^ Studies have suggested that inflammatory mediators in the TME, such as tumor necrosis factor-α (TNF-α), ceruloplasmin, and IL-8, can drive TANs to have a longer lifespan and greater immunosuppressive capacity than circulating neutrophils,^4,33–35^ included in the brain TME^34^ (Fig. 1). Subpopulations of TANs migrating initially to the tumor periphery frequently exhibit distinctive phenotypes, such as the transitional TAN-0 subset in non-small cell lung cancer (NSCLC) patients, migrating across the endothelium from the blood into the TME.^36^

## Phenotype of tumor-associated neutrophils

Strictly categorizing TANs into N1 and N2 phenotypes oversimplifies their complexity. The study identified 10 distinct neutrophil states across 17 human tumor types, including two tumor-specific subsets, pro-angiogenic vascular endothelial growth factor A (VEGFA)^+^ secreted phosphoprotein 1 (SPP1)^+^ TANs and antigen-presenting human leukocyte antigen (HLA)-DR^+^ CD74^+^ TANs, which are similar to subsets in the mouse TME.^37^ With the widespread application of Single-cell RNA sequencing analysis, immunofluorescence, immunohistochemistry staining, and multi-omics analysis in the study of TAN subtypes in tumors such as pancreatic ductal adenocarcinoma (PDAC),^38^ NSCLC,^39–41^ hepatocellular carcinoma (HCC),^42^ and gastric cancer,^43^ the complex and diverse gene expression, phenotypes and niche framework of TANs have been decoded (Fig. 1). Based on their roles in tumors and their maturation processes, they can be roughly classified into seven categories on the spectrum of transitions from an early classical or antitumor state to a protumor state in humans (Table 1). First, there is nonspecific TAN phenotype with the classical neutrophil feature, such as the and TAN-0 subclusters, as the researchers found in PDAC.^38^ TANs exhibit an immature antitumor phenotype in the early stage of tumors, such as the antigen-presenting human/mouse N_2_ phenotype in NSCLC discovered by Zilionis.^41^ With further interaction between TANs and the TME, TANs gradually develop a transitional TAN phenotype and mature into an immune-suppressive population that promotes tumor progression, including subtypes that promote angiogenesis,^39^ secrete myeloid chemokines,^42,43^ or produce inflammatory mediators.^39,40^ In addition, specific tumors possess unique phenotypes, such as lipid metabolism-related human/mouse apolipoprotein (APO) A2^+^ TANs found only in HCC.^42^ Of note, the IFN-activated TANs phenotype is not simply considered to have antitumor effects,^41–43^ as in the simple N1/N2 classification system. In special TME, IFN-activated TANs (such as interferon-induced tetrapeptide repeat (IFIT1)^+^ TANs in HCC) also exert a pro-tumor or uncertain effect.^42^ Therefore, the use of IFN^+^ TANs as therapeutic targets for tumors requires analysis of the specific TME conditions. Furthermore, other studies have revealed a “hybrid” TAN subgroup exhibiting APC-like features, with strong T-cell-stimulating activity, which may contribute to the antitumor effects of CD8^+^ cytotoxic T lymphocytes in early-stage human cancer (Fig. 1).^44,45^ It is closely related to IFN-γ and granulocyte-macrophage colony-stimulating factor (GM-CSF) levels and hypoxia in the TME.^45^

**Table 1:** Summary of marker systems for tumor-associated neutrophil subtypes in different tumors

The type of tumor	Pro-inflammatory subtype	Myeloid chemokine-secreting subtype	Proangiogenic subtype	Transitional subtype	IFN-stimulated subtype	Antigen presentation subtype	Classical subtype	^Refs^
PDAC	*NLRP3 CD69 PDE4B IL1RN ADM*			*S100A8 S100A9 MME VNN2 SELL*	*IFIT1 IFIT2 IFIT3 ISG15 RSAD2*			^38^
Tumor-draining lymph nodes in NSCLC	*CXCR3 CDH1 PLAC8*		*CXCL2 IL10 APOE*		*TNFRSF9 IL15 CCL19*	*HLA-DQB1 HLA-DPB1 CD1E*	*S100A8 S100A9 CTSG*	^39^
NSCLC	*CCL3 CCL4 CCL20 CCL3L1 CCL4L2 CXCL8 CXCL2 PI3 FNIP2 CSTB LGALS3 CSF1 IRAK1 IRAK2 MIF*		*PLIN2 LRPAP1*		*IFIT1 IFIT2 IFIT3 IFI6 MX1 RSAD2 ISG15 TDG CDK12 PRDX1 NOL8 DDX18 TRMT112 NARS HSP90AB1 HSP90AA1 HSPA1A HSPH1 CTSB MMP12 IRF7 OAS2*		*S100A8 S100A9 S100A12 PADI4 MMP9 ARG1 FCN1 CLEC4D*	^40,41^
Primary liver cancer		*CCL4 CCL4L2 CCL3 CCL3L1*	*SPP1 DYNLL1 LGALS3 PTMA S100A10*		*IFIT1 IFIT2 IFIT3 IFI6 MX1 RSAD2 HERC5 HES4*	*CD74 IGκC*	*LCN2 LTF MMP8 MMP9*	^42^
Gastric cancer		*CCL6 CD14 CD27 CXCL2 CD54 CCL5 CXCR2 EGR1 GDF15 CD54 ODC1 PWP1*			*CD63H CD81 CX3CR1 ISG15 MMP8 RSAD2*			^43^

*PDAC* pancreatic ductal adenocarcinoma, *NSCLC* non-small cell lung cancer, *IFN* interferon

However, due to the short lifespan,^18^ fragility, and low transcriptional capacity of TANs,^46^ which challenges the technology, many studies still rely on the N1/N2 status to describe TANs. Regarding neutrophil heterogeneity, some perspectives suggest it more likely reflects the transition in neutrophils at a certain maturation and/or reprogramming stage, as well as the potential for reprogramming TANs. For instance, recent work reveals that immature and mature neutrophils undergo deterministic reprogramming when infiltrating pancreatic tumors, converging into a distinct, terminally differentiated dcTRAIL-R1^+^ (T3) state predominantly localized at the tumor core.^47^ TANs in the dcTRAIL-R1^+^ state exhibit prolonged longevity and promote angiogenesis within hypoxic and glycolytic tumor niches to enhance tumor oxygenation and nutrition.^47^ This suggests that the protumoral N2 phenotype may represent an endpoint of sequential functional states during TAN polarization, originating from the N1 phenotype, thereby providing a potential avenue for targeted therapies based on reprogrammed TANs.

Moreover, immunosuppressive neutrophils, known as granulocytic/polymorphonuclear myeloid-derived suppressor cells (G/PMN-MDSCs), share similarities with N2 TANs.^48^ The obvious distinction between the two in tumor tissues remains the subject of debate^49,50^ (Fig. 2). Human PMN-MDSCs have been characterized in many studies as CD14^−^/CD15^+^/CD66b^+^/CD33^dim^/CD11b^+^/HLA-DR^−^ cells.^49^ Research has found that in the circulation of NSCLC patients, shared genetic signatures of mature PMN-MDSCs are significantly enriched in TANs, suggesting a striking similarity between human PMN-MDSCs and TANs at the transcriptional level.^51^ The circulating neutrophils of tumors exhibit heterogeneity and can be divided into low-density neutrophil (LDN) and high-density neutrophil (HDN) subgroups by density gradients.^52^ However, some researchers have pointed out that the term HDNs is inaccurately defined and may cause confusion because it refers to normal-density neutrophils (NDNs).^53^ LDN population comprises a blend of large mature neutrophils and immature neutrophils (akin to G/PMN-MDSCs).^52^ In tumor-bearing mice, circulating LDNs, which exhibit tumor-promoting and immunosuppressive properties, exhibited gradual augmentation when compared to tumor-free mice, contrasting with the decline observed in anti-tumor and cytotoxic NDNs.^52,54,55^ TANs can originate from NDNs and LDNs,^56^ with N1 and N2 phenotypes corresponding to NDNs and LDNs, respectively^52,57^; however, the correlation between them is still under debate. The polarization status of these cells is also regulated by factors such as IFN-β and TGF-β (discussed in “Tumor-suppressing Molecular Mechanisms of TANs”).^58^ The relationship between human LDNs and PMN-MDSCs is also closely intertwined. Current research suggests that immature LDNs correspond to PMN-MDSCs in murine tumors, whereas mature LDNs may correspond to circulating N2 type neutrophils (Nc2), though this remains controversial and researchers need to study it further to reach a consensus.^59^

## Tumor-promoting molecular mechanisms of TANs

**Fig. 3:**
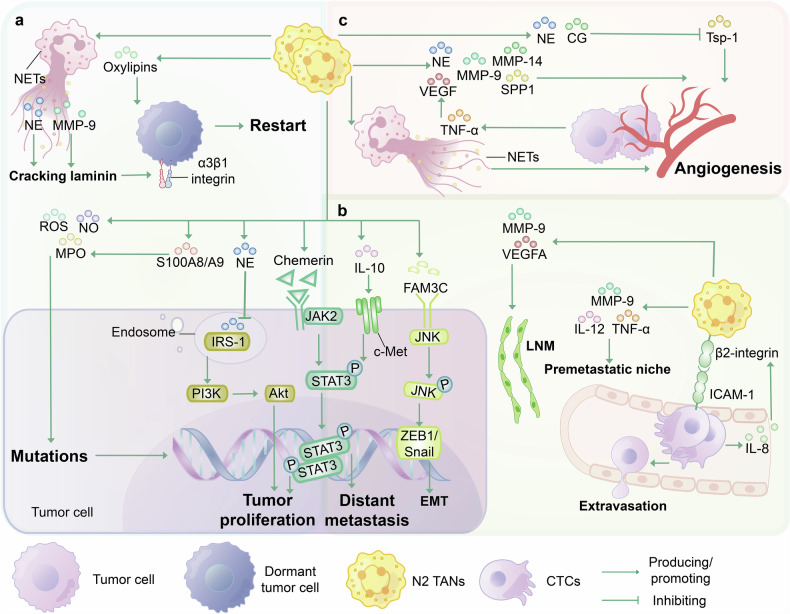
Tumor-associated neutrophil oncogenic mechanisms. TANs can promote tumor progression through many mechanisms, including inducing tumorigenesis, proliferation, and recurrence; enhancing tumor invasion and metastasis; and fostering angiogenesis. **a** Tumorigenesis, proliferation, and recurrence. TANs can expel NETs, wherein NE and MMP-9 can degrade laminin, activate α3β1 integrin signaling, or directly release oxylipins to awaken dormant cancer cells. The release of ROS, NO, and MPO by N2 TANs can induce DNA damage. The S100A8/A9 protein secreted by TANs can promote the release of MPO and oxylipins; therefore, tumor progression can be inhibited by targeting S100A8/A9. NE derived from TANs mediates the degradation of IRS-1 in the endosomes of tumor cells and promotes tumor proliferation by regulating the PI3K/Akt signaling pathway. **b** Tumor invasion and metastasis. TANs promote tumor cell proliferation and distant metastasis through the JAK2/STAT3 signaling pathway. N2 TANs can enhance distant metastasis and PD-L1 expression in tumor cells through the IL-10/c-Met/STAT3 signaling pathway, thereby promoting the polarization of TANs toward the N2 phenotype. FAM3C released by TANs promotes EMT through the JNK-ZEB1/Snail signaling pathway. The secretion of MMP-9, VEGFA, IL-12, and TNF-α in TANs can also promote LMNs and premetastatic niches. Tumor cells promote the expression of β2-integrin in neutrophils by secreting IL-8, which, in turn, binds to ICAM-1 and promotes the transendothelial migration of CTCs. One of the therapeutic directions is to target pathways that promote tumor metastasis via TANs. **c** Angiogenesis. TANs produce NETs and factors such as VEGF, NE, and MMP-9, accompanied by the hydrolysis of Tsp-1 by NE, to drive tumor angiogenesis. (Abbreviations: NE neutrophils elastase, NETs neutrophil extracellular traps, MMP matrix metalloproteinase, MPO myeloperoxidase, LNM lymph node metastasis, IRS-1 insulin receptor substrate-1, c-Met cellular-mesenchymal epithelial transition factor, JNK c-Jun N-terminal kinase, ZEB1 zinc finger E-box binding homeobox 1, Tsp-1 thrombospondin-1.)

Extensive research has reported that tumor-promoting TANs can promote tumor progression by facilitating tumor cell occurrence, proliferation and recurrence, invasion and metastasis, angiogenesis, and regulating the TME (to be discussed in detail later),^60^ which may provide new targets and perspectives for tumor-targeted therapy (Fig. 3).

### The occurrence, proliferation and recurrence of tumor cells

During chronic inflammation, immunosuppressive TANs induce DNA damage, increased DNA instability, and changes in the TME by producing and releasing ROS, nitric oxide (NO), and MPO and subsequently participate in malignant cell transformation.^61–63^ TANs can also alter tumor growth patterns and promote tumor proliferation by secreting metabolites and interacting with other cells. A study demonstrated that TAN-derived NE mediated the degradation of insulin receptor substrate-1 within tumor cells, thereby skewing the phosphoinositide 3-kinase (PI3K) axis toward tumor proliferation.^64^ Chemerin can regulate TANs and activate the Janus kinase (JAK) 2/signal transducer and activator of transcription (STAT) 3 pathway in oral squamous cell carcinoma (OSCC) cells in vitro, which upregulates downstream signaling targets including phosphorylated retinoblastoma (pRb) protein, E2F transcription factor 1 (E2F1), cell cycle protein E1 (Cyclin E1), and cell cycle protein D1 (cyclin D1), thereby promoting tumor cell proliferation and metastasis.^65^

After colonizing surrounding tissues, tumor cells can enter a dormant state, thereby ceasing proliferation or growth and evading immune surveillance while maintaining metabolic activity.^66,67^ Studies have shown that TANs can reverse the dormant phenotype of some dormant cancer cells and restart proliferative programs in vivo.^68,69^ The role of NETs was investigated in promoting tumor arousal. In a mouse breast cancer model, the sustained inflammatory environment in the lungs induced by lipopolysaccharides promoted neutrophils to expel NETs overlaid with NE and matrix metalloproteinase (MMP)-9. Proteases on NETs induced the awakening of dormant cancer cells through remodeling laminin and initiating matrix and activating integrin α3β1 signaling.^70^ Subsequently, in a dormant model of lung and ovarian cancer, stress hormones stimulated PMN-MDSCs to secrete substantial amounts of the proinflammatory S100A8/A9 protein, further inducing MPO activation and the accumulation and release of oxylipins; this in turn upregulated fibroblast growth factor and induced the awakening of dormant tumor cells.^71^ In summary, TANs can trigger the generation and proliferation of tumor cells, and restart the dormant tumor cells (Fig. 3a).

### Tumor invasion and metastasis

TANs can facilitate the metastasis of various cancers, such as lung cancer,^72^ CRC, breast cancer,^73^ and gastric cancer, to the lungs, liver, and lymph nodes through various mechanisms, such as the release of NETs.^73–76^ Metastatic tumors also exhibit a significant enrichment of TANs, making TAN infiltration a predictive factor for tumor metastasis.^77^ Recently, emerging evidence has suggested that N2 TANs potentiate distant metastasis and programmed cell death ligand-1 (PD-L1) expression in human/mouse lung cancer cells through the IL-10/cellular-mesenchymal epithelial transition factor/STAT3 signaling pathway.^78^ The STAT3/PD-L1 axis promotes the polarization of TANs to the N2-like state, thus forming a positive feedback loop.^78^ Next, we summarize various molecular mechanisms by which TANs activate tumor invasion and metastasis (Fig. 3b).

Metastasis requires the induction of premetastatic niche formation at a distant site before tumor cells reach the target organ, which prepares tumor cells for long-distance transmission and colonization.^79^ Tumor-derived factors (such as CXCL1 and CXCL12) activate and recruit TANs into the premetastatic niche to play an important role in suppressing or supporting metastasis.^80^ Subsequently, TANs release proinflammatory factors, such as cytokines (TNF-α and IL-12) and the protease MMP-9, to facilitate premetastatic niche formation and the homing of tumor cells.^81^ The invasion-metastasis cascade encompasses the processes of local invasion, intravasation, and extravasation of cancer cells and the formation and cloning of micrometastases,^82,83^ with TANs playing an important role in protumor migration. Melanoma cells promote neutrophil β2-integrin expression by producing and secreting IL-8.^84^ In turn, neutrophil β2-integrin binds to intercellular adhesion molecule 1 on melanoma cells, tethering circulating tumor cells (CTCs) to the endothelium of the target organ and promoting tumor migration across the endothelium.^84^

Epithelial-mesenchymal transition (EMT) refers to the process wherein epithelial cells undergo the loss of epithelial features, such as cell polarity and intercellular junctions, and acquire mesenchymal-like phenotypic traits, including increased motility and invasiveness.^85^ EMT significantly influences tumor invasion, metastasis, and drug resistance.^86^ Studies have shown that tumor cells induce TANs through TGF-β1, which activates the Smad 2/3 signaling pathway, leading to the activation of the family with sequence similarity (FAM) 3C, a cytokine-like gene in the FAM3 family.^87^ FAM3C facilitates tumor cell EMT and subsequently promotes lymph node metastasis (LNM) in gastric cancer through the JNK-ZEB1/Snail signaling pathway^87^ rather than through the AKT pathway, as described in other studies.^88,89^ FAM3C may be considered an innovative biomarker or promising target for cancer therapy.^87^ Moreover, TANs can also regulate the JAK2/STAT3 signaling pathway through chemokines, which can not only facilitate tumor proliferation as mentioned earlier, but also induce EMT in OSCC and promote the migration and invasion of tumor cells.^65^

The methods used to detect tumor metastasis include LNM, hematogenous dissemination, and implantation metastasis. Most epithelial tumors initially colonize local lymph nodes, circulate in the lymphatic system, and then spread through the blood. Therefore, LNM is very important for promoting tumor dissemination. Neutrophil clusters of tumor cells within the lymphatic vessels of gastric cancer patients were identified a propensity for lymph node metastasis.^87^ As previously described, TANs can promote LNM through the tumor cell EMT pathway.^87^ TANs can also increase the number of channels for tumor cell invasion by promoting lymphangiogenesis,^90^ enhancing the survival of tumor stem cells, and suppressing antitumor immunity, thus promoting lymphatic metastasis and tumor invasion.^91^ Research has revealed that the transcription factor ETS variant 4 can enhance the transcription of CXCL1/8 in bladder cancer cells to promote the recruitment of TANs, leading to increased secretion, bioavailability, and bioactivity of MMP-9 and VEGFA in TANs.^92^ Additionally, this process stimulates the formation of inflammatory human lymphatic endothelial cell tubes, further facilitating the migration and invasion of bladder cancer cells.^92^

### Angiogenesis

A functional vascular system can support solid tumor growth and metastasis. The formation of neovascularization in tumors involves the sprouting of the endothelium and the recruitment of pericytes. In mice, the hypoxic and glycolytic TME can reprogram TANs to adopt a pro-angiogenic phenotype.^37,47^ As previously discussed, a specific neutrophil phenotype characterized by VEGFA^+^ SPP1^+^ TANs is identified, which also possess pro-angiogenic capabilities.^37^ Previous studies have reported that the pro-angiogenic properties of TANs are associated with TNF-α^13^ and a lack of type I IFN,^93^ and drive tumor angiogenesis and vascular by producing angiogenic factors such as VEGF and proteases, including NEs and matrix MMPs,^94–96^ especially MMP-9.^58,97,98^ Many tumors express TNF-α, which induces the degranulation of TANs to release VEGF, which promotes endothelial proliferation and angiogenesis.^13^ NE degrades and remodels the extracellular matrix,^94,95^ and during inflammation, neutrophils can also hydrolyze the antitumor factor thrombospondin-1 via the NE and CG, promoting angiogenesis and ultimately lung metastasis.^99^ Recent studies have shown that TAN-derived SPP1 (osteopontin/OPN) and MMP-14 can promote endothelial cell migration and vascular branching, respectively.^100^ Moreover, in human and murine gastric cancer, neutrophils are prone to release NETs, which bind to NET-DNA receptor CCDC25 on endothelial cells, thereby promoting endothelial proliferation, survival, and migration.^101^ Consequently, NETs can exert VEGF-like effects, facilitating tumor angiogenesis and increased microvascular density,^101^ holding potential as novel targets for anti-angiogenic therapy (Fig. 3c).

## Tumor-suppressing molecular mechanisms of TANs

**Fig. 4:**
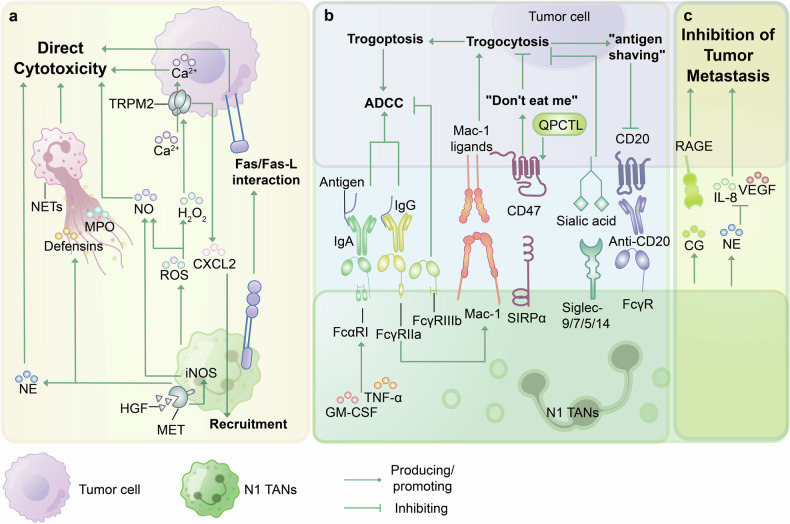
Tumor-associated neutrophil tumoricidal mechanisms. TANs exert antitumor effects through mechanisms such as direct cytotoxicity, antibody-opsonized killing, and inhibition of tumor metastasis. **a** Direct cytotoxicity. TANs directly kill tumor cells by releasing NE, NO, and H_2_O_2_ or forming NETs to produce MPO and defensins. The HGF/MET pathway can induce the production of iNOS and NO in TANs. H_2_O_2_ can activate TRPM2, leading to Ca^2+^ influx to kill tumor cells. TRPM2 also enhances the expression of CXCL2, promoting additional TAN recruitment. Moreover, the Fas/Fas-L interaction is implicated in the promotion of cytotoxic mechanisms. **b** Antibody-opsonized killing. Fc receptors on the surface of TANs interact with the Fc domains of antibodies bound to cancer cells, mediating ADCC. Mac-1 enhances FcγR-mediated trogocytosis, which is inhibited by the CD47-SIRPα axis and Siglec-9/7/5/14-sialic acid interactions. Therefore, using antibodies or blocking the CD47-SIRPα axis to promote TAN ADCC against tumors is another therapeutic approach. Trogocytosis mediates the removal of the target antigen CD20 from tumor cells. Enhancing the trogocytosis function of TANs against tumors and suppressing resistance are currently therapeutic avenues. **c** Inhibition of tumor metastasis. CG and NE produced by TANs can inhibit the migration of tumor cells. (Abbreviations: CG cathepsin G, iNOS inducible nitric oxide synthase, Mac-1 macrophage-1 antigen, RAGE receptor for advanced glycation end products, HGF hepatocyte growth factor.)

Early studies have demonstrated the potential of neutrophils to kill tumor cells both in vitro and in vivo.^14,102^ TANs exert antitumor effects through direct cytotoxicity, antibody-mediated killing, inhibition of tumor metastasis, and stimulation of the immune TME (to be discussed in detail later) (Fig. 4).

### Direct cytotoxicity

Antitumor TANs can directly kill tumor cells by producing ROS, other cytotoxic mediators and proteases, and NETs, as well as increasing the expression of Fas (Fig. 4a).^2,103^

ROS/reactive nitrogen species, which are products of activated neutrophils, consist of several key components, including single oxygen (^1^O_2_), superoxide (O_2_·^−^), hydrogen peroxide (H_2_O_2_), hydroxyl radical (HO^−^), NO, and peroxynitrite (ONOO^–^).^104^ Upon reaching tumor cells, TANs secrete H_2_O_2_ through contact-dependent mechanisms, activating the H_2_O_2_-dependent calcium (Ca^2+^) channel, namely, the transient receptor potential channel melastatin 2 (TRPM2),^105^ which induces a lethal influx of Ca^2+^ to restrict the formation of metastases.^54^ Moreover, TRPM2 can increase the expression of CXCL2, a key ligand of CXC-chemokine receptor (CXCR) 2, which is crucial for the recruitment of TANs.^106^ Although tumor cells generally express higher levels of TRPM2 to facilitate normal growth, in heterogeneous tumor cell populations, certain cells may express lower levels of TRPM2 (either through knockout or knockdown), thereby downregulating CXCL2 expression in tumor cells and blocking the Ca^2+^-dependent apoptotic cascade to evade the cytotoxic effects of TANs.^106^ Moreover, tumor-derived TNF-α or other inflammatory stimuli activated the *Met* proto-oncogene on neutrophils in mice and humans, promoting the recruitment of TANs to the TME and neutrophil induction of inducible nitric oxide synthase (iNOS) under hepatocyte growth factor (HGF, the ligand of MET) stimulation.^107^ This cascade enabled TANs to generate NO, thereby slowing tumor growth and metastasis. However, the status of host immune cells and the adaptation of tumor cells to oxidative stress influence the net tumor-regulatory effects mediated by ROS production in neutrophils. Li et al. demonstrated that G-CSF was amplified in host NK cell-deficient mice and that TANs suppressed the spread of breast tumor cells in the lung, while in mice with active NK cells, TANs promoted metastasis.^108^ They found that ROS produced by neutrophils not only inhibit the antitumor activity of NK cells but also kill tumor cells.^108^ Therefore, the ROS generated by TANs represent a double-edged sword, with their specific impact on tumors depending on the composition of the TME and the immune evasion capabilities of tumor cells. This might explain why certain subtypes of IFN-activated TANs (such as IFIT1^+^ TANs) do not necessarily exhibit the anti-tumor characteristic of N1 TANs.^42^

A study showed that in humans, rather than murine PMN-MDSCs, catalytically active NEs can be released to kill genetically diverse cancer cells while retaining noncancer cells selectively.^109^ However, the experiment did not verify the selectivity of the antitumor effect of human TANs and whether it is primarily mediated by NE. Therefore, further research is needed on the role of NE produced by TANs on tumors, although it is now generally accepted that the role of NE in the TME in humans is biased towards a pro-tumor effect. Furthermore, in a mouse model of pancreatic adenocarcinoma, melatonin induces recruitment and fatty acid oxidation of TANs, which activates TANs into an N1-like antitumor phenotype and generates a higher level of ROS in the TME.^110^ ROS mediate the release of NETs via an NADPH oxidase 2-independent pathway, leading to the apoptosis of tumor cells through MPO and defensins or cell-to-cell contact, a mechanism that may also be present in humans.^24,110^ We hypothesize that the difference in the effect of TANs-produced NETs on the tumor may be related to the precise location of TANs in the tumor and the difference in the period of the tumor and TANs; For example, NETs released by N1 TANs often act at the edge of the tumor and exert antitumor effects, while NETs produced by N2 TANs are often located in the center of the tumor, near dormant cancer cells, or the vasculature, thus playing the role of promoting matrix lysis and angiogenesis to promote tumor.^111^

Moreover, N1 TANs can counteract metastasis formation by generating cytotoxic substances (Fig. 4c). For instance, neutrophil CG can recognize tumor cells by binding to the receptor for advanced glycation end products on tumor cells,^112^ exerting antitumor cytotoxicity and inhibiting tumor cell metastasis.^113^ In addition, in the inflammatory site, activated neutrophils can produce angiostatin and NE, which degrade angiogenic factors such as IL-8, basic fibroblast growth factor, and VEGF in a time- and concentration-dependent manner, leading to the deprivation of angiogenic activity.^114^ However, the TME and inflammatory microenvironment often exhibit stark differences, thus the specific phenotypes and mechanisms through which TANs inhibit angiogenesis remain elusive.

### Antibody-opsonized killing properties of TANs

As early as 1975, Gale and Zighelboim demonstrated the role of neutrophils in eradicating antibody-activated tumor cells in vitro through antibody-dependent cell cytotoxicity (ADCC).^115^ The fragment antigen-binding domains of therapeutic antibodies can bind to tumor-associated antigens on the surface of tumor cells, while their fragment crystallizable (Fc) regions can bind to Fc receptors (FcRs) on neutrophil surfaces, thus mediating the recognition between neutrophils and antibody-opsonized tumor cells, facilitating tumor cell killing through mechanisms such as ADCC (Fig. 4b).^116^ Neutrophil IgG-bound FcRs, include FcγRIIa (CD32a), FcγRIIIb (CD16b), and FcγRI (CD64), which are expressed only after activation; FcγRIIc (CD32c), which is expressed at low levels in some populations^117^; and FcαRI (CD89), which binds to IgA. Due to its high binding affinity, FcγRI promotes tumor killing in many types of tumors,^118,119^ but no effect was observed when monovalent Fc fragments were used to block FcγRI. This may be due to the inability of these monovalent Fc fragments to block the receptor completely or to their relatively low expression levels on neutrophil surfaces.^120^ In antibody therapy, high expression of FcγRIIIb decreases the availability of therapeutic antibodies, restricts signal transduction, and negatively regulates neutrophil-mediated ADCC.^121^ In a transgenic mouse model expressing FcαRI, cytokines such as GM-CSF and TNF-α induce FcαRI expression, which is highest in neutrophils.^122^ FcαR1 has a greater affinity for IgA antibodies and is considered the most effective neutrophil FcR for activating ADCC.^123^ However, the short half-life of IgA hinders its activity in vivo.^124^

Neutrophils have developed a mechanism different from other immune cell-mediated cytotoxic mechanisms known as trogocytosis, which is involved in neutrophil-mediated ADCC. This process (referred to as trogoptosis), in which neutrophils endocytose cytoplasmic fragments of tumor cells, resulting in the dissolution (i.e., necrosis) of antibody-opsonized tumor cell, relies on the interaction between the macrophage-1 antigen (Mac-1) and FcγR (especially FcγRIIa).^125^ Interestingly, during rituximab treatment of B-cell malignancies, trogocytosis mediates the removal of the target antigen CD20 from tumor cells, thus averting the increased sensitivity of cancer cells to destruction by anti-CD20 antibodies and generating a protumorigenic drug-resistant effect.^126^ The dominant view holds that the mechanism underlying this phenomenon is that trogocytic cells selectively capture plasma membrane proteins from donor cells, most likely through the direct binding of the plasma membranes of the two cells or the exchange of the intermediate vesicles at the contact points.^127^ However, researchers found that TANs could not kill tumor cells opsonized with Anti-EGFR (epidermal growth factor receptor) Abs of the IgA isotype through antibody-dependent trogocytosis in the immunosuppressive TME of NSCLC patients,^128^ which may be related to the levels of tumor cell antigen expression and effector-to-tumor cell ratios in the TME.^128^ Thus, future therapeutic strategies utilizing TANs’ antitumor effects may hinge on conditions that enhance trogocytosis mechanisms favorable to TANs in the TME.^128^

Furthermore, due to the overexpression of checkpoint molecules, the efficacy of targeted antibody-mediated immunotherapy and the ADCC function of neutrophils are diminished. For example, The “Do not eat me” signal formed by the binding of the CD47 protein, which is expressed on the tumor cells, to signal-regulatory protein alpha (SIRPα), which is expressed on myeloid cells, enables tumor cells to evade immune surveillance. Glutaminyl-peptide cyclotransferase-like protein (QPCTL) is a major component of the CD47/SIRPα checkpoint.^116,129^ However, despite the potential enhancement of IgA-mediated neutrophil ADCC through inhibition of the CD47/SIRPα axis or QPCTL function, tumor cells exhibit resistance to immunotherapy using targeted therapeutic antibodies, suggesting the existence of additional myeloid checkpoint molecules.^130–132^ A recent study has identified that malignant tumor cells overexpress α2,3-linked sialic acids, which primarily recognize and bind sialic acid-binding immunoglobulin-like lectin 9 (Siglec-9) and a small subset of Siglec-7 on neutrophils in vitro and in vivo mouse models.^130^ Both the CD47/SIRPα axis and the sialic acid/Siglec-9 axis signal through immunoreceptor tyrosine-based inhibitory motifs (ITIMs) and/or ITIM-like domains within neutrophils, ultimately activating downstream signal pathways that inhibit neutrophil ADCC.^132^ In an in-vivo transplanted mouse model, blocking the interaction between Siglec-9 expressed on neutrophils and its ligands expressed on EGFR and HER2-positive breast tumor cells enhances IgG1 or IgG2 antibody-driven neutrophil ADCC against EGFR and HER2.^130^ Furthermore, recent research indicates that Siglec-5/14 also inhibits neutrophil-mediated ADCC in tumor cells.^133^ Hence, a combined strategy to concurrently block sialic acid/Siglecs (particularly Siglec-9) and CD47/SIRPα has a potential to improve targeted antibody-based tumor immunotherapy and overcome tumor resistance.^131^

## Crosstalk between TANs and the TME

**Fig. 5:**
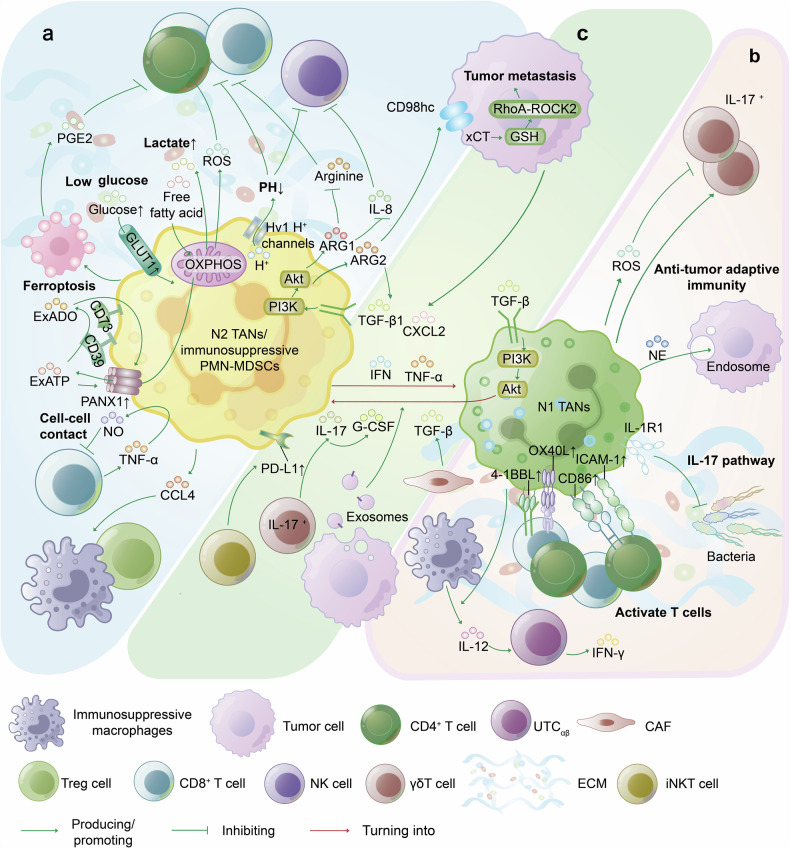
Interactions of tumor-associated neutrophils with other cells in the tumor immune microenvironment. **a** Immunosuppressive TME. TANs recruit immunosuppressive macrophages and Treg cells via chemokines such as CCL4. The mechanisms through which TANs impair T cells include cell-cell contact, the generation of ROS (metabolic transition from OXPHOS to free fatty acid-dependent OXPHOS), and ferroptosis-induced PGE_2_ release. The Hv1H^+^ channel on TANs promotes acidification of the TME, enhancing tumor cell survival while impairing the antitumor functions of T cells and NK cells. TANs with high expression of PANX1 and CD39/CD73 collaboratively construct an immunosuppressive TME with exADO. The secretion of ARG1 and ARG2 by TANs can inhibit the activity of T cells and NK cells and promote cancer cell metastasis, respectively, suggesting the potential therapeutic application of ARG1 inhibitors in tumor treatment. **b** Immunostimulatory TME. TANs stimulate macrophages to produce IL-12, enhancing the antitumor effects of UTC_αβ_. The upregulation of the costimulatory molecules ICAM-1, CD86, OX40L, and 4-1BBL on TANs also stimulates T-cell responses and proliferation. TANs can modulate the IL-17 pathway, limiting bacterial colonization to suppress protumor inflammatory responses. NE and ROS released by TANs enhance adaptive antitumor immunity and suppress IL-17^+^ γδT cells, respectively, accompanied by the activation of antitumor γδT cells. **c** Phenotypic transition of TANs. IFN, TNF-α, tumor cell-derived CXCL2, and inhibition of TGF-β can induce N1 polarization of TANs. Conversely, TGF-β, IL-17, G-CSF, and tumor cell-derived exosomes can induce N2 polarization. Targeting TAN reprogramming through strategies such as anti-TGF-β therapy is a promising approach in cancer therapy. (Abbreviations: ECM extracellular matrix, OXPHOS oxidative phosphorylation, GLUT1 glucose transporter type 1, PANX1 Pannexin 1, GSH glutathione, CD98hc heavy chain of CD98, xCT system x cystine/glutamate antiporter, RhoA Ras homolog gene family member A, ROCK2 rho-associated coiled-coil-containing protein kinase 2, IL-1R1 IL-1 receptor type 1, UTC_αβ_ CD4^−^CD8^−^αβ T cell.)

The crosstalk between tumor cells and immune cells creates a unique TME that can both promote tumor growth, invasion, and metastasis and inhibit tumor progression.^134^ The role of TANs in the TME remains controversial. Numerous studies have suggested that TANs are induced by different TME conditions, regulate their metabolism and interactions with other immune cells, and contribute to the formation of either immune-suppressive or immune-stimulatory TMEs through extracellular matrix remodeling. Conversely, the TME can also reprogram and modulate the phenotype and function of TANs (Fig. 5).

### The immunosuppressive TME

The characteristics of TME hypoxia, high levels of ROS, high lactate levels, changes in hormone levels, and low potential for hydrogen (pH) further reprogram TANs to promote the progression of the immunosuppressive TME (Fig. 5a). Under low glucose conditions, TANs undergo a metabolic shift from oxidative phosphorylation (OXPHOS) to free fatty acid-dependent oxidative phosphorylation, which results in the production of damaging ROS that impair CD4^+^ T cells and increase glucose uptake and lactate production in both tumor cells and TANs, contributing to the immunosuppressive TME.^135–137^ The upregulation of glucose transporter type 1 expression and increased glucose metabolism in TANs promote tumor growth and attenuate the effect of radiotherapy.^138^ Dysregulation of redox mechanisms can lead to excessive peroxidation of polyunsaturated phospholipids, causing nonapoptotic forms of ferroptosis.^139^ In the TME, ferroptosis of PMN-MDSCs induces the release of oxidized lipids (such as prostaglandin E2 (PGE_2_)) and the accumulation of oxidized arachidonic acid-phosphatidylethanolamines, limiting the activity of human and murine T cells and inducing the transformation of nonsuppressive neutrophils into immunosuppressive PMN-MDSCs.^139^ However, in cisplatin-mediated NSCLC, ferroptosis-induced N1 TANs exhibit antitumor effects.^140^ TANs in mouse tumors express voltage-gated Hv1 H^+^ channels, which promote the acidification of the TME by modulating the intracellular pH, enhancing the survival and mobility of tumor cells, and impairing the antitumor function of T and NK cells.^141^ Under physiological conditions, Pannexin 1 mediates the release of intracellular ATP as extracellular ATP (exATP),^142^ which promotes immune responses. In the TME, exATP is metabolized by nucleotidases into extracellular adenosine (exADO), which suppresses local immunity.^143^ In basal-like breast cancer, TANs with high expression of CD39/CD73 were positively correlated with high expression of Pannexin 1, leading to TAN recruitment and the formation of an immunosuppressive TME with high exADO.^144^ In amino acid-related metabolic pathways, TANs are the main cells that secrete ARG1 and ARG2.^145^ Studies have shown that the annexin A2/TLR2/myeloid differentiation factor 88 pathway induces the secretion of ARG1 by TANs in NSCLC, which in turn depletes arginine to suppress T-cell viability and reduces IL-18 secretion to suppress NK cell activation, thereby promoting an immunosuppressive TME.^145–147^ In the CRC TME, CRC cells actively recruit TANs through CXCL2 and produce TGF-β1, which induces peripheral blood neutrophils to transform into AGR2^+^ TANs by triggering the PI3K-AKT signaling pathway.^148^ Secreted AGR2 activates the glutathione-Ras homolog gene family member A-rho-associated coiled-coil-containing protein kinase 2 cascade through its receptor heavy chain of CD98-system x cystine/glutamate antiporter, promoting CRC metastasis.^148^ Additionally, AGR2 increases TGF-β1 expression, forming a positive feedback loop between CRC cells and AGR2^+^ TANs stimulated by TGF-β1.^148^

In addition, the release and function of NETs in the TME are associated with the aging status of neutrophils connected to circadian fluctuations, and the stimulation in TME such as hypoxia and tumor-related factors (e.g., CXCL2).^19,110,149^ A recent study discovered that chronic stress in mice disrupts the circadian rhythm of neutrophils by releasing glucocorticoids (GCs) and induces NETosis, thus promoting a microenvironment conducive to the pulmonary metastasis of disseminated cancer cells.^150^ The GC signal alters the expression of the key clock gene *Per1* in neutrophils, leading to an aberrant diurnal aging phenotype, increased neutrophil counts in blood and tumors, and NETs release.^150^ Synchronized with the abundance of immunosuppressive MDSCs, the delivery of immune checkpoint inhibitors showed the most effective outcomes, highlighting the potential role of neutrophil circadian rhythms in optimizing the timing of cancer therapy and potentially enhancing anti-tumor immune efficacy.^19,151^

N2 TANs also engage with other immune cells such as CD8^+^ T cells,^42,152–154^ NK cells,^155^ and TAMs^156^ through various mechanisms and recruit regulatory T cells, thereby shielding tumor cells from CD8^+^ T cell- and NK cell-mediated cytotoxicity and promoting an immunosuppressive TME.^156–158^ PD-L1^+^ TANs can inhibit the proliferation and activation of CD8^+^ T cells in some tumors.^42,152,153^ A study demonstrated a correlation between NO release by TANs isolated from tumors in vivo and TNF-α paracrine signaling by T cells. In turn, TANs kill nonactivated T cells through NO in a contact-dependent manner, thereby inhibiting CD8^+^ T-cell antitumor activity.^154^ Neutrophils can also promote the intraluminal survival of trapped tumor cells by suppressing NK cell function.^155^ In intrahepatic cholangiocarcinoma, TANs and TAMs exert synergistic effects on tumor growth and metastasis within the TME, facilitated by direct cell-cell contact or soluble mediators and surpassing the individual pro-tumoral effects of TANs or TAMs alone.^156^ Co-culture experiments revealed heightened production of oncostatin M by TANs and IL-11 by TAMs, both activating STAT3 signaling in tumor cells.^156^ In both mouse and human tumors, TANs recruit immunosuppressive macrophages and regulatory T (Treg) cells through chemokine signaling pathways, such as the CCL4-C-C motif chemokine receptor 5 pathway.^42,134,157^

### The immunostimulatory TME

N1 TANs can effectively shape the TME formation and promote antitumor immunity by regulating innate and adaptive immunity (Fig. 5b). NE released by N1 TANs can be absorbed by breast cancer cells, which sensitizes them to cyclin E-CD8^+^ cytotoxic T lymphocyte-mediated cytolysis and enhances the recognition of antitumor adaptive immunity.^159^ Bacteria in the murine TME can induce protumorigenic neutrophils such as the main periodontal pathogen *Porphyromonas gingivalis*, upon colonization in pancreatic cancer tissue, promoting a TAN-predominant inflammatory TME and facilitating tumor progression.^160^ Conversely, IL-1 receptor type 1 on neutrophils regulates IL-17-mediated inflammatory responses, limiting bacterial colonization and suppressing inflammation-induced tumor cell proliferation and DNA damage.^161,162^ However, the mechanisms by which bacteria influence the interaction between TANs and TME remain elusive.

Moreover, N1 TANs interact with T cells,^4,163,164^ macrophages,^165^ NK cells,^166^ and other immune cells through various cytokines, chemokines, and proteases to establish an antitumor cellular network. As mentioned earlier, neutrophils endowed with antigen presentation capabilities can upregulate HLA and costimulatory molecules to activate T cells,^167^ such as the HLA-DR^+^CD74^+^ neutrophil subset identified.^167^ In addition, CCL4^+^ TANs also were discovered to recruit macrophages in HCC, while PD-L1^+^ TANs suppress T cell cytotoxicity.^42^ In the early stages of lung cancer, TANs stimulate T-cell responses and proliferation.^4^ The interaction between activated TANs and T cells results in the upregulation of the expression of the costimulatory molecules intercellular adhesion molecule 1, CD86, OX40L, and 4-1BBL on neutrophils, which promotes T-cell proliferation via a positive feedback loop and IFN-γ release.^4^ Additionally, coculturing activated γδ T cells with autologous neutrophils enhances their antitumor effects on PDAC cells.^168^ However, IL-17^+^ γδ T cells (γδ17 T cells) promote tumor growth and metastasis while expressing low levels of the antioxidant glutathione, increasing susceptibility to neutrophil-derived ROS.^169^ TANs strongly inhibit the proliferation of γδ17 T cells through the TAN/ROS/γδ17 T-cell axis.^169^ In an in vitro coculture model, TANs promoted IL-12 production by macrophages, resulting in the type 1 polarization of the atypical CD4^−^CD8^−^αβ T-cell (UTC_αβ_) subpopulation.^165^ Subsequently, CD4-CD8-αβ T-cells produce IFN-γ to exert an antitumor effect in vivo.^165^ Furthermore, in a mouse model of CRC undergoing autologous hematopoietic stem cell transplantation, researchers identify neutrophil infiltration within tumors, which enhances NK cell activation and suppresses their activation-induced death during homeostatic proliferation.^166^ TANs, along with T cells, macrophages, NK cells, and other components establish a robust antitumor cellular network within the TME, paving the way for developing effective strategies for TAN-related antitumor immunotherapy.

### TME-mediated phenotypic conversion of TANs

The protumor or antitumor phenotypic plasticity of TANs can be modulated by signals presented by various cells in the TME (Fig. 5c).^170^ Coculture with tumor cells can induce a wide range of gene expression changes in which neutrophil TANs enter an active state, promoting tumor progression and activating protein synthesis.^171^ In the TME of glioblastoma, the function of neutrophils was found to be successfully reprogrammed by tumor cells after 72 h of contact. In addition, the plasticity of TANs occurs in response to the stimuli they receive and depends on the duration of the TME.^172^ Therefore, signaling pathways that reshape TANs in the TME could serve as new targets for cancer therapy. IFN,^58,173^ TNF-α,^174^ and blockade of TGF-β can induce N1 polarization.^103^ In contrast, TGF-β,^2^ IL-17,^175^ and G-CSF can induce N2 polarization.^176^ Furthermore, the source of TANs, NDNs, exhibits tumor-induced phenotypic plasticity, which can be induced by tumor-regulated mediators and CXCL1 to transition to a low-density state.^56^ The latter acquire the morphological, functional activity, and surface receptor expression characteristics of LDNs, indicating that LDNs may be partially derived from phenotype-changing NDNs^56^ (Fig. 2).

#### TGF-β

TGF-β, a common cytokine in tumors, is often one of the main cytokines in pathways that induce N2 TANs^177^ and further inhibit the antitumor function of T cells and NK cells.^178^ Blocking TGF-β activity has been shown to induce TANs to polarize toward the N1 phenotype and impede the progression of tumors such as CRC,^103^ NSCLC,^179^ HCC,^180^ and mesothelioma.^4^ Cancer-associated fibroblast (CAF)-derived cardiotrophin-like cytokine factor 1 (CLCF1) can enhance the expression of CXCL6 and TGF-β in tumors, leading to TAN recruitment and N2 polarization in the middle and late stages, respectively.^180^ Therefore, selectively blocking cardiotrophin-like cytokine factor 1 signaling may provide a new method for treating HCC patients.^180^ Anti-TGF-β treatment can partially inhibit the PI3K/AKT signaling pathway in TANs, promoting their polarization toward an antitumor phenotype and reducing the secretion of metastatic chemoattractants.^103^ Additionally, TGF-β blockade remarkably increased the expression of GM-CSF and IFN-γ in SW480 cells (a human CRC cell line) cocultured with TANs, which enhanced the polarization of TANs to the N1 phenotype and promoted their antitumor effects.^103^ The mechanisms by which TGF-β inhibition polarizes TANs toward an antitumor N1 phenotype and increases their cytotoxicity also include elevated ROS levels.^103^ Furthermore, evidence that Smad3 induces TANs to polarize to the predominant N2 phenotype in lung cancer through the TGF-β1/Smad3 signaling pathway, while Smad3 deficiency induces polarization to the N1 phenotype, has been reported.^181^ Gene deletion and drug inhibition of Smad3 may enhance its anti-NSCLC ability by promoting the polarization of TANs to the N1 state.^181^

#### IFN and other signal pathways

In mice and humans, IFN promotes the polarization of TANs toward the antitumor N1 phenotype and enhances their ability to kill tumor cells directly.^58^ A study performed in mouse models lacking endogenous IFN showed that compared with mice with sufficient IFN, mice with TANs exhibited reduced production of ROS, and these TANs demonstrated significantly decreased cytotoxicity.^182^ A reduction was reported in tumor growth in mouse models with β-glucan-induced trained immunity due to the reprogramming of neutrophils toward an antitumor phenotype.^183^ This process requires type I IFN.^183^ Mechanistically, in the presence of IFN-γ and TNF-α, TANs restored the activation of the PI3K and p38 mitogen-activated protein kinase pathways, decreased the expression of the *Bv8* and *Mmp9* genes and increased the levels of IL-18 and NK-activating ligands.^174^

In the TME, factors (such as IL-17^184^) and derivatives (such as exosomes^185,186^) exist to reshape the phenotype of TANs through various signaling pathways,^185,187^ predominantly associated with the formation of N2 TANs, highlighting the prevalent immune-suppressive milieu orchestrated under tumor cell dominance. For instance, IL-17 participates in promoting neutrophil recruitment and differentiation toward an immunosuppressive phenotype.^184^ In a mouse model of spontaneous breast cancer metastasis, IL-1β has been shown to induce IL-17 expression in γδ T cells, leading to systemic G-CSF-dependent expansion and polarization of neutrophils toward a CD8^+^ T-cell-suppressive phenotype.^175^ Additionally, exosomes derived from gastric cancer cells and CRC stem cells can drive neutrophil polarization toward the N2 subtype.^185,186^ In a mouse CRC model, iNKT cells have reduced respiratory burst capacity, inducing the expression of PD-L1 and promoting its inhibitory effect on CD4^+^ T-cell proliferation to affect the activation state of neutrophils and promote an immunosuppressive phenotype.^188^ Despite numerous factors inducing the formation of N2 TANs, as previously described, pancreatic melatonin can induce tumor cells to secrete CXCL2, regulating TANs toward an N1-like antitumor phenotype.^110^

## Potential applications of TANs in tumor therapy

Tumor immunotherapy influences the therapeutic efficacy and prognosis of cancer by modulating the immune defense function of immune cells against tumors and reshaping the TME. However, the strength of its effect across different types of tumors remains controversial. Current immunotherapies mainly include checkpoint blockade and adoptive cell transfer therapy. The combination of tumor immunotherapy with traditional cancer treatments can generate synergistic antitumor responses.^189^ In recent years, various TAN-related tumor immunotherapies, targeted therapies, chemotherapies, and combination therapies have received extensive attention as promising therapeutic strategies. Numerous emerging studies have evaluated the therapeutic efficacy and safety of utilizing TAN-related therapies in cancer treatment. In this section, we will discuss the various strategies, current status, and future directions of utilizing TANs in tumor therapy (Table 2).

**Table 2:** Summary of preclinical studies and clinical trials of TAN-targeted cancer therapy

Class of target	Agents	Tumor type	Alone or in combination	Effects of therapy in preclinical studies	Clinical stage	Clinicaltrails.gov	^Refs.^
Clinical trials							
CXCR2 inhibitor	AZD5069	Prostate cancer	AZD5069+enzalutamide		I/II	NCT03177187	^206^
	AZD5069	Hepatocellular carcinoma	AZD5069 + durvalumab		I/II	ISRCTN12669009	^253^
CXCR1/2 inhibitor	SX-682	Metastatic castration-resistant prostate cancer	SX-682 + enzalutamide		II	NCT06228053	^207^
	SX-682	Metastatic colorectal cancer	SX-682 + TriAdeno vaccine + retifanlimab + N-803		I/II	NCT06149481	^208^
	SX-682	Pancreatic cancer	SX-682 + tislelizumab		II	NCT05604560	^209^
	SX-682	Metastatic or recurrent stage III C or IV NSCLC	SX-682 + pembrolizumab		II	NCT05570825	^210^
	SX-682	Metastatic colorectal cancer	SX-682 + nivolumab		I/II	NCT04599140	^211^
S100A9 inhibitors	Tasquinimod	Metastatic castrate resistant prostate cancer	Alone		II	NCT01732549	^217^
NE inhibitors	Fisetin	Childhood cancer	Alone		II	NCT04733534	^226^
CD47/SIRPα	Hu5F9-G4	B-cell non-Hodgkin’s lymphoma	Rituximab or rituximab + chemotherapy		I/II	NCT02953509	^235^
ARG1 inhibitor	INCB001158	Solid tumor	Alone or INCB001158 + pembrolizumab		I/II	NCT02903914	^237^
TGF-β inhibitors	SAR-439459	Malignant solid neoplasm	SAR-439459 + cemiplmab		I	NCT04729725	^245^
TREM1 agonist	PY159	Solid tumor	Alone or PY159 + pembrolizumab		I	NCT04682431	^251^
Preclinical studies							
CXCR2 inhibitor	SB225002	Nasopharyngeal carcinoma	SB225002+radiotherapy	After 18-day treatment, compared with the control group, mean decreased tumor volume (≥300 mm^3^), tumor weight (≥150 mg), and TAN accumulation (≥10%)			^202^
	SB225002	Lung adenocarcinoma and squamous cell carcinoma	Alone or SB225002+cisplatin	Compared with the control group, significant reduction of TAN infiltration (alone: 18.85%) and elevated activated CD8^+^ T lymphocytes (alone: 35.13%)			^203^
	AZD5069	NASH-HCC	Alone or AZD5069 + anti-PD-1	At day 28, compared with the control group, decreased tumor burden (AZD5069: ≥500 mm^3^, AZD5069 + anti-PD-1: ≥1500 mm^3^); the accumulation and reprogramming of TANs			^252^
CXCR2/1 inhibitor	SCH-479833	Pancreatic cancer	Alone	After 21-day treatment, compared with the control group, mean decreased tumor weight (≈ 1000 mg) in mice bearing CD18/HPAF-xenograft tumors			^204^
Bcl-xL inhibitor	A-1331852	Lung adenocarcinoma and squamous cell carcinoma	Alone or A-1331852 + G-CSF	On average, tumors in the control group doubled their size after 2 weeks, whereas tumors in treated mice were on average 1.3 times bigger compared to their size before treatment with a reduced proportion of total TANs			^35^
CTC-neutrophil clusters	GV-Lipo/sorafenib/DT	HCC	Alone	The blockade of CTC-neutrophil clusters and reduction of CTCs in mice; inhibition rate of recurrence and metastasis: 95.42%, H22-bearing tumor model: 90.28%, orthotopic HCC model: 99.81%			^223^
CD20+ HER2	X-body (rituximab + trastuzumab)	Lymphoma and solid tumor	Alone	Increased toxicity of neutrophils, NK cells, and macrophages to tumor cells; infiltration and activation of NK cells and neutrophils; after 21-day treatment, compared with the control group, reduction of tumor volume (≥1000 mm^3^) in mice			^231^
EGFR	TrisomAb	Colorectal cancer	Alone	Recruited NK cells, macrophages, and neutrophils as effector cells; after 18-day treatment, compared with the control group, reduction of tumor volume (≈900 mm^3^) in mice			^124^
CD47/SIRPα	VK30	Lung cancer	Alone	Reduction of G-MDSC infiltration (≥10%), promotion of macrophage phagocytosis index(＞ 6%), and apoptosis of tumor cells in mice			^232^
HDAC inhibitor	CN133	PCa	CN133 + anti-PD-1	At day 25, the subcutaneous PCa mice models: reduction of PMN-MDSC infiltration in the blood (10%) and anti-tumor positive TME			^238^
TGF-β1 receptor inhibitor	SB525334	Pancreatic cancer	SB525334 + IRE + anti-PD-1	The infiltration of neutrophil, CD8^+^ T cells, the polarization of TAN anti-tumor phenotype, reduction of tumor volume (≥1300 mm^3^) at week 9, and the enhancement of immunotherapy in mice			^243^
STAT5	GTN	Triple-negative breast cancer	GTN + doxorubicin	The enhancement of doxorubicin’s antitumor effect, the recruitment and activation of CD8^+^ T cells, and the inhibition of the immunosuppressive function of PMN-MDSCs in mice			^254^

*ARG1* arginase 1, *Bcl-xL* B-cell lymphoma-extra large, *CTC* circulating tumor cell, *CXCL* CXC-chemokine ligand, *CXCR* CXC-chemokine receptor, *DT* digitoxin, *EGFR* epidermal growth factor receptor, *G-CSF* granulocyte colony-stimulating factor, *GTN* glyceryl trinitrate, *HCC* hepatocellular carcinoma, *HDAC* histone deacetylase, *HER2* human epidermal growth factor receptor 2, *IRE* irreversible electroporation, *mAb* monoclonal antibody, *NASH* non-alcoholic steatohepatitis, *NE* neutrophil elastase, *NSCLC* non-small cell lung cancer, *PCa* prostate cancer, *PD-1* programmed cell death-1, *SIRPα* signal-regulatory protein alpha, *STAT* signal transducer and activator of transcription, *TGF-β* transforming growth factor β, *TREM1* receptor expressed on myeloid cells-1, *X-body* X-shaped antibody

### Inhibition of the tumor-promoting effect of TANs

#### Targeting chemokine

Within the TME, TANs exist in various states, with N2 TANs being predominant. Therefore, a comprehensive understanding of the factors driving the recruitment of different states of TANs and the selective depletion of protumor TANs by targeting the binding of chemokine ligands and their receptors can effectively inhibit tumor growth. Furthermore, selective depletion of pro-tumoral subtypes of TANs can alter the phenotypic composition of TANs, contributing to the suppression of tumor growth. Mimicking the depleted human pro-tumor TAN subtypes (Neu_09_IFIT1, Neu_10_SPP1, and Neu_11_CCL4) selectively by using an anti-ly6G antibody in a mouse model of liver cancer, the study found that the anti-tumor mouse Neu_09_Apoa2 subtype (corresponding to human Neu_07_APOA2) expanded within tumors, macrophage recruitment was hindered and T cell was suppressed, thereby restraining tumor progression.^42^

CXC chemokine ligands (such as CXCL1,^190^ 5,^191,192^ and 6^193^), G-CSF,^27^ IL-17,^188^ and TGF-β can promote cancer cell invasion and metastasis by recruiting TANs.^194^ The recruitment of TANs can further enhance this signaling pathway.^195^ Numerous studies have shown that neutrophils express the highest levels of CXCR2 among blood cells, suggesting that the ligands CXCL1 and CXCL8/IL-8 are crucial chemotactic agents for N2-type TANs.^196^ The CXCR2/CXCL8 axis regulates the recruitment,^26^ NETosis, and lymphatic metastasis of TANs in various cancers,^92^ such as breast cancer^197^ and HCC.^26^ It has been suggested that there may be a positive feedback loop in which TANs secrete CXCL8, which then recruits neutrophils to polarize into TANs.^158^ Upregulation of CXCL1 also promotes tumor growth by promoting TAN invasion in lung cancer and melanoma.^190^ However, a study revealed that the levels of CXCL1 and CXCL8 were not associated with the number of TANs in gastric cancer.^198^ Other studies have reported that knocking down CXCR2 in mouse breast cancer led to increased TAN infiltration and a more pronounced pro-tumor N2 TAN spectrum, favoring tumor growth and lung metastasis.^199^ Moreover, brain metastatic variants of the breast cancer cells can also activate CXCR2 receptors on TANs and promote recruitment in tumor regions and a pro-NETotic state of TANs.^200^ Therefore, the functional complexity of the CXCR2 axis in TANs across different types of tumors requires further investigation.

In preclinical studies, the CXCR2 antagonist SB225002 was shown to exert significant antitumor effects by inhibiting N2 TAN recruitment. For instance, in papillary thyroid carcinoma tissues, tumor cells produce CXCL8 to recruit TANs.^201^ In immune-deficient mice, administration of the CXCR2 antagonist SB225002 can significantly eliminate TAN accumulation and retard tumor growth, showing promising antitumor effects.^201^ SB225002 can also be combined with radiotherapy to inhibit angiogenesis and radiation-induced TAN recruitment in a nasopharyngeal carcinoma model in vitro and in vivo, demonstrating potent antitumor activity.^202^ In preclinical studies, SB225002 has shown similar antitumor efficacy in lung adenocarcinoma and squamous cell carcinoma, as well as enhancing the therapeutic effects of cisplatin.^203^ Furthermore, in preclinical models, the CXCR2/1 antagonist SCH-479833^204^ and traditional Chinese medicine (such as the Yi Qi Chu Tan Formula)^205^ can also suppress neutrophil recruitment, tumor cell proliferation, migration, and angiogenesis. In clinical trials, the CXCR2 antagonist AZD5069 (Study NCT03177187)^206^ and the CXCR1/2 antagonist SX-682 (Study NCT06228053,^207^ Study NCT06149481,^208^ Study NCT05604560,^209^ Study NCT05570825,^210^ Study NCT04599140^211^ and so on), which are also associated with TAN recruitment, have shown increasing clinical benefits in antitumor treatment.^205^

However, due to the pleiotropic effects of factors such as CXCL8 and the phenotypic diversity of TANs, caution must be exercised when testing CXCR1/2 inhibitors to prevent unexpected toxicity.^212^ Most studies indicate that CXCR1/2 inhibitors are generally safe, but some clinical trials have revealed that CXCR2 inhibitors, such as AZD5069, can lead to reduced blood neutrophil counts in patients with chronic obstructive pulmonary disease.^213^ Therefore, achieving optimal dosing and duration of CXCR2 inhibitors is crucial for effective management. In addition, the antiapoptotic B-cell lymphoma-extra large (Bcl-xL) protein prolongs the lifespan of protumorigenic TANs via JAK/STAT signaling, with tumor cell-derived GM-CSF triggering the expression of Bcl-xL.^35^ In a mouse model, the use of the Bcl-xL inhibitor A-1331852 selectively can reduce the abundance of protumor TANs, replacing them with antitumor TANs, with the administration of G-CSF counteracted neutropenia in patients.^35^ Therefore, targeting alternative pathways that selectively inhibit N2 TANs also represents a promising direction for future cancer therapies.

#### Targeting S100A8/A9

S100A8 and S100A9 are Ca^2+^-binding proteins belonging to the S100 family and are mainly expressed in neutrophils and monocytes, forming a common heterodimeric structure, S100A8/A9.^214^ As discussed above, S100A8/A9 can serve as a target to suppress TAN-mediated tumor recurrence and eliminate the reactivation of dormant tumor cells induced by stress.^71^ Furthermore, Zhang et al. employed scRNA-seq on human PDAC and hepatic metastase tissues to dissect the subpopulations of neutrophils within the TME.^215^ They identified two predominant neutrophil clusters in the metastatic TME, namely cluster 4 (SLPI neutrophils) and cluster 8 (S100A8 neutrophils).^215^ Notably, S100A8 was highly expressed in the metastatic TME, accompanied by elevated expression of cluster 8 marker genes such as CXCL8 and nicotinamide phosphoribosyltransferase, which suggested a close association between S100A8 produced by neutrophil clusters in the TME and tumor metastasis.^215^ For instance, in a phase II clinical trial involving patients with metastatic castrate-resistant prostate cancer, the S100A9 inhibitor tasquinimod demonstrated significant therapeutic efficacy (Study NCT01732549).^216,217^

However, studies have shown that S100A8/A9 is crucial for the development of the gut microbiota and immune system in mice and postnatal infants,^218^ as its knockout has been found to cause embryonic lethality in mice.^219^ Therefore, directly targeting S100A8/A9 for antitumor purposes may have some adverse side effects. Although S100A8/A9 has pro-tumor effects, evidence suggests that high concentrations of S100A8/A9 can paradoxically inhibit tumor growth.^220^ For instance, overexpression of S100A9 can trigger apoptosis in NB4 cells (acute promyelocytic leukemia cells).^220^ Moreover, S100A8/A9 participates in complex networks of signaling pathways, and mechanistic studies are largely confined to theoretical research. Annotations regarding its functions remain incomplete, necessitating further validation through clinical trials. A study discovered that CD300ld was specifically expressed and upregulated on PMN-MDSCs after tumor occurrence; these cells recruited PMN-MDSCs into tumors through the STAT3-S100A8/A9 axis and inhibited T-cell activation.^221^ Interestingly, the deletion of CD300ld did not affect the development of mice and could synergistically inhibit tumor growth in conjunction with anti-programmed cell death-1 (PD-1) treatment.^221^ Therefore, combining drugs targeting key components of the S100A8/A9 signaling pathway with various antitumor therapies may represent a promising treatment strategy in the future.

#### Targeting angiogenic factor

Based on the aforementioned underlying mechanisms, targeting CTC-neutrophil clusters can inhibit the promotion of the tumor invasion-metastasis cascade by TANs. Neutrophils can interact with CTCs through vascular cellular adhesion molecule-1 (VCAM-1) to form CTC-neutrophil clusters.^222^ Inhibiting the binding of VCAM-1 prevents their formation.^222^ For instance, a study developed a multipoint costriking nanodevice (GV-Lipo/sorafenib/digitoxin (DT)) that utilized an anti-VCAM-1 monoclonal antibody to target, capture and prevent the formation of CTC-neutrophil clusters and used DT to dissociate the CTC clusters to enhance cancer treatment efficacy in preclinical model.^223^

Currently, other inhibitors targeting key factors (such as NE and VEGF) in TANs that promote tumor metastasis are also being tested in clinical trials. Chemotherapy prophylactic drugs, flavonoid compounds, can serve as NE inhibitors in human neutrophils.^224,225^ For example, fisetin (a flavonoid compound) is undergoing phase II clinical trials in adult survivors of childhood cancer (study NCT04733534^226^). Furthermore, by isolating pro-angiogenic TANs from IFN-deficient mouse tumor models and disrupting the signaling cascade of nicotinamide phosphoribosyltransferase (NAMPT) associated with VEGF production within isolated TANs, it is possible to reprogram TANs towards an anti-angiogenic phenotype.^93^ Local transfer of these reprogrammed TANs into tumors could reduce the toxic side effects of systemic application of inhibitors targeting VEGF and other related signaling pathways and precisely attack tumor cells.^93^ However, this emerging approach currently remains more feasible in mice, and further exploration is required to validate its application in human experiments and clinical settings.

Side effects of anti-angiogenic therapy targeting VEGF/vascular endothelial growth factor receptor (VEGFR) 2 include an increased likelihood of developing resistance in breast cancer patients.^227,228^ The resistance mechanisms involve high-dose anti-VEGFR2 treatment with the drug apatinib, which triggers IL-17A expression in γδ T cells through activation of the VEGFR1-PI3K-AKT pathway, which in turn promotes N2-like TAN differentiation and CD8^+^ T cell exhaustion.^227^ Apatinib combined with immunotherapy targeting IL-17A, PD-1, or Ly-6G monoclonal antibodies (mAbs) can target the immune regulatory axis of “γδ17 T-cell-N2 TANs” in vivo, reshaping the apatinib-induced immunosuppressive TME and alleviating resistance.^227^

### Enhancement of the antitumor effect of TANs

Exploiting the powerful antibody-mediated killing properties of TANs represents a promising direction for cancer immunotherapy. Clinical studies involving TANs in ADCC have demonstrated that TANs can modulate adaptive immune responses to establish long-term antitumor immunity in the treatment of cancer with mAbs.^229^ Most of the antitumor mAbs used clinically are IgG isotypes.^230^ However, the binding of IgG isotype mAbs to FcγRI is competitively blocked by endogenous IgG saturation in vivo, and these mAbs cannot induce TAN migration.^124^ There is a lack of sufficient evidence to demonstrate the prominent role of TANs in IgG-based tumor immunotherapy.^229^ Thus, recent preclinical studies have synthesized an X-shaped antibody (X-body) that combines the activity of both IgG and IgA.^231^ In addition, in another preclinical study, IgG mAbs and FcαRI bind to therapeutic bispecific antibodies (or TrisomAb molecules), which both recruit and activate NK cells, macrophages, and neutrophils to clear tumor cells.^124^ Both TrisomAbs and X-bodies had longer half-lives than IgA in vivo and significantly reduced tumor growth with stronger toxicity against tumor cells than did IgG.^124^

Immune checkpoint blockade is a treatment in which drugs are used to block the binding of a pair of immune checkpoints, thereby inhibiting the ability of tumor cells to “shut down” the immune system and combat cancer. Regarding the mechanism by which TANs kill antibody-activated tumor cells, current preclinical evidence suggests that neutrophil-mediated ADCC can be increased by blocking the CD47-SIRPα axis, increasing the efficacy of antibodies targeting tumors.^129^ At present, several antibodies or therapeutic biological agents targeting CD47 or SIRPα have been developed in clinical settings. CD47-targeted drugs include anti-CD47 antibodies and fusion proteins composed of the N-terminal IgV domain of SIRPα and SIRPα-Fc.^116^ Recent preclinical studies have demonstrated the antitumor effects of the novel CD47-blocking peptide VK30, which reduces the infiltration of N2 TANs and G-MDSCs in tumor tissues as well as their expression of tumorigenic IL-6 and TNF-α.^232^ Furthermore, breast cancer cells were found to evade neutrophil-induced trogocytosis via Ca^2+^-dependent and exocyst complex-dependent plasma membrane repair.^233^ Knocking out either of these vesicle components, exocyst complex components (EXOC) 7/4, can reduce tumor cell resistance to neutrophil-mediated ADCC.^233^ As previously described, neutrophil trogocytosis may cause “antigen shaving” by reducing surface CD20 expression on target B lymphoma cells, potentially leading to widespread resistance to rituximab or anti-CD20 therapy.^126^ However, sodium stibogluconate and CD47-SIRPα blockade (necessary and insufficient conditions) can convert neutrophil trogocytosis of rituximab-conditioned malignant B cells into cell killing.^126^ This occurs primarily through FcγRI rather than FcγRIIa, likely because FcγRI mediates the killing of B cells by these anti-CD20 conditioning proteins.^126^ Clinical trial in phase Ib/II demonstrates favorable effects of antibodies targeting the CD47-SIRPa checkpoint (Study NCT02953509).^234,235^ Moreover, when the neutrophils first reach the tumor sites and the TME is in a state of immune activation, killing tumor cells through trogocytosis may be a way to kill tumor cells in the cradle successfully.^31^ Therefore, reports on the complex anticancer effects of TANs, such as neutrophil trogocytosis, remain insufficient and have mostly been obtained from in vitro experiments or mouse models. However, further research is needed to determine their therapeutic potential.

### Targeting the interaction between TANs and the TME

Based on the previous discussion on the amino acid metabolic pathway by which TANs promote an immune-suppressive TME, the ARG1 inhibitor INCB001158 has been reported to reverse the immune-suppressive effects of TANs. A phase I/II clinical trial has been conducted to evaluate the efficacy of INCB001158 alone or in combination with pembrolizumab (anti-PD-1) for the treatment of metastatic solid tumors (Study NCT02903914).^236,237^ In a preclinical murine subcutaneous prostate cancer model, the histone deacetylase (HDAC) inhibitor CN133 combined with anti-PD-1 inhibited the infiltration of soft prostate cancer tissue by PMN-MDSCs and significantly reduced the secretion of enzymes (ARG1 and iNOS) by PMN-MDSCs that suppress CD8^+^ T cells.^238^ On the other hand, although ferroptosis of PMN-MDSCs promotes an immune-suppressive TME, clinical trials more commonly use ferroptosis inducers to directly induce tumor cell death and harness the antitumor effects of TANs.^139^ Inducing tumor cell ferroptosis by cysteinase (depletion of cystine or cysteine) in mice sensitized tumor cells to IFN-γ and synergized with immune checkpoint blockers to inhibit tumor growth.^139,239^

Furthermore, in a murine colon cancer model, a novel therapeutic approach combines photodynamic therapy (PDT) with oncolytic bacterial immunotherapy (OBI).^240^ OBI involves engineered bacteria specifically accumulating in tumor tissues, promoting the formation of immunostimulatory TME and killing tumor cells, albeit potentially associated with side effects such as infections.^240,241^ In this study, PDT is a therapy that kills cancer cells and bacteria through the generation of ROS by laser-activated photosensitizers (PSs) and the corresponding light source.^242^ Researchers use the engineered *S. typhimurium* to deliver photosensitizers, which produce ROS to destroy tumor cells and bacteria, releasing PAMPs and DAMPs that recruit and activate neutrophils in large numbers.^240^ Newly recruited neutrophils exhibit robust anti-tumor activity, release TNF-α and IL-1β to further promote immune cell infiltration and the formation of immunostimulatory TME, and counteracts bacterial overgrowth to prevente potential side effects from excessive bacterial presence.^240^ Thus, the OBI-PDT combination therapy, by leveraging multiple roles of TANs in anti-tumor activities, bactericidal effects, and immune cell recruitment, demonstrates broad application prospects of the targets involved in interaction between the TME and TANs.

### Reprogramming of TANs

The precise role of TANs in the treatment response remains to be determined; thus, simple inhibition or promotion of TAN function is not sufficient for tumor therapy. It is crucial to exploit the phenotypic switch mechanism of TANs to restore their antitumor function and continuously modulate cytotoxic N1 TANs to exert their function within the target.

TGF-β inhibitors can regulate neutrophil polarization toward an antitumor N1 phenotype.^243^ Currently, the combination of TGF-β inhibitors such as SAR-439459 and the PD-1 inhibitor cemiplimab is being tested in a phase 1b trial (study NCT04729725).^244,245^ However, systemic administration of inhibitors targeting TGF-β or its receptors may induce toxic effects that affect cardiac development and function.^246^ For example, inhibitors of TGF-βRI such as AZ12601011 and AZ12799734 have been shown to impact cardiac valve integrity in rat models.^246^ The irreversible electroporation (IRE) ablation technique in pancreatic cancer combined with PD-1 immune checkpoint blockade has shown promising efficacy and induced extensive infiltration of TANs into tumors. By locally utilizing silicon dioxide nanoparticles loaded with the TGF-β1 receptor inhibitor SB525334 in the TME, IRE-induced polarization of recruited TANs into an antitumor N1 phenotype can be facilitated in preclinical study.^243^ Furthermore, it could enhance the response of pancreatic cancer to IRE and PD-1 combination therapy and induce long-term antitumor memory.^243^ However, further research is needed to study its side effects.

Recombinant IFN-α2, the first FDA-approved immunotherapy for treating human solid and hematologic malignancies.^247^ In addition, TLR agonists and STING agonists are potent inducers of type I IFN. However, systemic administration of recombinant type I IFN sometimes comes with side effects, such as hepatotoxicity and promotion of autoimmune inflammatory diseases.^248,249^ Targeting the triggering receptor expressed on myeloid cells-1 (TREM1) expressed on TANs can lead to the production of factors such as IFN-γ, further promoting the antitumor phenotype of neutrophils.^250^ Currently, the TREM1 agonist PY159 is being evaluated alone or in combination with pembrolizumab to assess its safety and efficacy in patients with standard treatment-resistant and refractory solid tumors in a Phase I clinical trial (Study NCT04682431).^250,251^

In a mouse model of HCC with non-alcoholic steatohepatitis (resistant to anti-PD-1 therapy), the study found that a combination of the CXCR2 antagonist AZD5069 and anti-PD-1 therapy could reprogram intratumoral TANs to an anti-tumor phenotype^252^; And were organized in Granzyme B immune clusters (containing CD8^+^T cells), accompanied by increased activation of XCR1^+^ dendritic cells and increased numbers of CD8^+^ T cells in tumors, overcoming resistance to anti-PD-1 Therapy.^252^ Based on scRNAseq data, they hypothesized that CXCR2 antagonists preferentially inhibit mature TANs (which express high levels of surface CXCR2), while immature TANs with low PD-L1 expression can potentially overcome resistance to anti-PD-1 therapy. Clinical trials targeting CXCR2 in combination with durvalumab (PD-L1/PD-1 axis) in late-stage HCC phases I/II are currently underway (Study ISRCTN12669009).^253^ The ability of CXCR2 antagonists to reprogram TANs and the aforementioned evidence that melatonin regulates TANs via CXCL2, a ligand of CXCR2, to induce an N1-like phenotype may reflect a complex link between CXCL2/CXCR2, TANs, and melatonin.^110^ The combination of glyceryl trinitrate and doxorubicin-induced ROS-dependent STAT5 lysis in PMN-MDSCs, accompanied by a reduction in fatty acid transport protein 2 (FATP2), which reprogrammed PMN-MDSCs to a less immunosuppressive phenotype.^254^ It also increased the recruitment of CD8^+^ T cells and PD-L1^low^ PMN-MDSCs within the tumor, enhancing the antitumor effect of capecitabine.^254^ Surprisingly, although the expression of *cxcr2* genes decreased, the number of PMN-MDSCs increased in the treated mice.^254^ Furthermore, lipofermata, by targeting FATP2 on PMN-MDSCs, reduced tumor size in mouse models.^255^ Currently, some emerging preclinical therapies, such as oncolytic viruses or therapeutic tumor vaccines based on acidic/photosensitive dendritic cells,^256,257^ reshape N2 TANs into N1 TANs, adjusting the TME to inhibit tumor growth. The combination of innovative therapies and TAN phenotypic remodeling holds great potential and prospects in the field of tumor treatment.

### Drug delivery carriers

In contrast to the strategies above depleting neutrophils or directly targeting TAN-related signaling pathways or mechanisms within the body, the neutrophil delivery strategy involves isolating circulating neutrophils, subjecting them to brief, transient stimuli to polarize towards an anti-tumor phenotype or loading them with drugs in vitro, and subsequently reintroducing them in vivo to target tumor cells.^37^ Such a therapeutic method first minimizes interference from the TME on neutrophil phenotype, leverages the short half-life of neutrophils to significantly reduce side effects, and effectively facilitates drug penetration across the blood-brain barrier.^39^

Due to the metalloproteinase expression, chimeric antigen receptor (CAR)-neutrophils can infiltrate into a denser tumor stroma compared to CAR-T cells.^258^ Human neutrophils have emerged as promising drug delivery vehicles due to their ability to efficiently cross the blood-brain tumor barrier and their enhanced safety profile. Recent preclinical studies have constructed CAR-neutrophils with optimal antitumor activity that are loaded with the hypoxia-activated prodrug tirapazamine.^259^ In combination with TANs, these agents can maintain the antitumor N1 phenotype and overcome the reprogramming of TANs in the TME.^259^ The specific and noninvasive delivery and release of nanodrugs targeting glioblastoma multiforme by CAR neutrophils reduced drug off-target effects and prolonged the lifespan of tumor-bearing mice.^259^ In another preclinical study, a drug delivery nanoplatform based on neutrophils and monocytes/macrophages was constructed, enhancing the tumor-targeting ability of neutrophils as drug carriers.^260^ Recently, a nanovehicle composed of an activated neutrophil membrane-incorporated liposome also succeeded in modulating the TME in a tumor xenograft mouse model.^261^ Similarly, recent studies have used neutrophils as carriers to deliver attenuated *Salmonella typhimurium* (VNP20009), allowing VNP to safely and accurately exert its anti-tumor effects on the tumor core.^262^ Using an engineered strain secreting the PD-1 nanobody, the TME can be further reshaped as antigen-presenting TME.^262^ However, current research still needs to address challenges such as the short lifespan of neutrophils, their susceptibility to apoptosis in vitro, their resistance to genome editing, and the reduction of neutrophils in vivo caused by their extraction. Furthermore, the accuracy with which chemotherapy drugs are targeted by neutrophil delivery also needs to be improved to reduce drug accumulation in the spleen, side effects on other organs, and even systemic toxicity.

On the other hand, neutrophil-derived exosomes (NDEs) have shown promise as carriers for various cargos, including lncRNAs, miRNAs, and cytotoxic proteins. Due to their natural biogenesis process, NDEs exhibit greater biocompatibility, circulation stability, and long-range targeting than traditional synthetic delivery vehicles.^263^ An NDE-like nanovesicle system from neutrophils loaded with doxorubicin and modified with superparamagnetic iron oxide nanoparticles was developed, which specifically targeted tumor sites and exerted antitumor effects.^264^ However, NDEs have a limited lifespan, low production productivity, and dual effects on tumors.^185,186,264^ In the future, it is important to consider how to modulate the effects of NDEs on tumors and overcome their limitations to engineer NDEs for precise targeting of tumor cells with chemotherapy drugs.

## Prognostic significance of TANs

In the TME, a high density of infiltrating neutrophils has emerged as a novel prognostic indicator for poor prognosis in most cancer types, such as lung cancer,^265^ oral cancer,^266,267^ and liver cancer.^268^ In patients with HCC, a higher TAN density is associated with elevated serum C-reactive protein (CRP) levels and can be combined with the serum C-reactive protein level to predict poor patient prognosis.^269^ In addition, the expression of the autoimmune regulator in N2 TANs is associated with poor prognosis in breast cancer tissue.^270^ However, in patients with gastric and esophageal adenocarcinoma, it was found that CD66b^+^ TANs were significantly associated with better prognosis in these cancer types and showed sex specificity—only in females.^271^ Another study on gastric cancer patients receiving neoadjuvant/perisurgical treatment also revealed sex-specific differences in TAN density.^272^ Apart from sex, the location of TANs, stage, and type of tumor also play important roles in the prognostic implications of TAN density.^273^ As mentioned earlier, TANs at the tumor-infiltrating edge often present as the N1 phenotype, while those in the tumor center tend to be the N2 phenotype. This may be related to the fact that the combined parameters of low intratumoral TANs, high stromal TANs, and high necrosis have been shown to indicate significantly greater 3-year disease-free survival in CRC patients.^273^ Due to the complexity of TAN subpopulations, there is still a lack of sufficient research on how to accurately describe TAN subpopulations and quantify TAN infiltration using reliable biomarkers to predict tumor prognosis based on TAN density.^274^ A study designed an activatable semiconducting polymer nanoprobe that utilized fluorescence and photoacoustic imaging techniques to image NE secretion from TANs in real time for cancer immunotherapy, thereby enabling the evaluation of the number of TANs in tumors, monitoring the response to cancer immunotherapy and predicting prognosis.^274^ However, derivatives of TANs in the TME may not accurately reflect the actual abundance of TANs, thus necessitating their specific identification with neutrophil markers such as CD66b.^59^

Moreover, in the circulation of cancer patients, the peripheral blood neutrophil-to-lymphocyte ratio (NLR) increases, making NLR a focal point in current research for assessing tumor prognosis due to its evaluability through blood tests.^274–276^ The NLR is a well-defined biomarker associated with poor prognosis in patients with tumors such as glioblastoma,^274^ NSCLC,^275^ and CRC.^276^ In circulation, neutrophils can secrete tumor-promoting factors such as IL-6, associated with poor prognosis. Consequently, the pro-tumor effects of circulating neutrophils (such as PMN-MDSCs) might be one of the reasons for the close association between NLR and adverse outcomes.^275^ Furthermore, the functional plasticity of peripheral neutrophils in tumors is evident, with G-CSF involved in mobilizing immature LDNs.^62^ For instance, in OSCC patients, LDNs show significantly increased IL-17 expression compared to NDNs, and the LDNs/NDNs ratio is also considerably higher compared to healthy people.^277^ This highlights the need for more detailed studies on peripheral neutrophils to analyze their impact on prognosis.^277^ Many neutrophil-related mediators in the circulation of cancer patients, such as NETs, are also associated with poor tumor prognosis and have been identified as independent prognostic factors in cancer.^70,101,278^ In breast cancer, the formation of NETs promotes lung metastasis, leading to poor prognosis.^278^ TANs and their associated indicators will become a potential focus in the field of tumor prognosis prediction in the future. Moreover, despite ongoing controversies regarding the discrimination of PMN-MDSCs from N2 TANs, PMN-MDSCs and their associated factors (such as CD300ld) are similarly closely associated with poor survival rates in the tissues and circulation of various tumors,^221^ such as bladder cancer.^279^

## Conclusions and perspective

Despite the previously overlooked role of TANs as innate immune cells in cancer, advancements in technology (such as single-cell analysis techniques) have increasingly recognized the importance of TANs in cancer therapy. In this review, we focus on (1) the dual role of various TAN phenotypes in tumors and (2) the TAN-TME interactions and phenotypic transition mechanisms of TANs. Based on these mechanisms and recent preclinical and clinical data, we analyzed the potential applications of TANs in five aspects of tumor therapy (inhibition of the tumor-promoting effect of TANs, enhancement of the antitumor effect of TANs, targeting the interaction between TANs and the TME, reprogramming of TANs, and drug delivery carriers) and their relevant key targets. Additionally, we briefly discuss the close association between TANs and the prognosis of patients receiving tumor treatment.

However, the controversy surrounding the classification of TAN phenotypes hinders a deeper understanding of the potential applications of TANs. Currently, the research and application of emerging technologies to distinguish TAN subsets and assess TAN infiltration in the TME will pave the way for the unified nomenclature of TAN subpopulations, the prediction of therapeutic efficacy, and further exploration of the molecular mechanisms underlying the role of TANs. Moreover, although many studies have successfully investigated numerous novel therapeutic targets in cell line xenograft mouse models or in vitro, reliable data from other mouse models and human models is still lacking. Therefore, more research and attention are needed in other tumor models to establish a connection between the molecular mechanisms by which TANs work and their potential clinical applications for the prognosis prediction of human patients. As research on TANs continues to progress, we anticipate that the potential of TANs in tumor therapy will benefit more cancer patients in the future.
